# Incidence and risk factors for infection in oral cancer patients undergoing different treatments protocols

**DOI:** 10.1186/1472-6831-12-22

**Published:** 2012-07-20

**Authors:** Manju Panghal, Vivek Kaushal, Sangeeta Kadayan, Jaya Parkash Yadav

**Affiliations:** 1 Department of Genetics, M. D. University, Rohtak, Haryana, India; 2 Department of Radiotherapy, Regional Cancer Institute, Pt. B.D.S, Health University, Rohtak, Haryana, India

## Abstract

**Background:**

Over the past decade, advances in cancer treatments have been counterbalanced by a rising number of immunosuppressed patients with a multitude of new risk factors for infection. Hence, the aim of this study was to determine risk factors, infectious pathogens in blood and oral cavity of oral cancer patients undergoing different treatment procedures.

**Methods:**

The present prospective cohort analysis was conducted on the patients undergoing treatment in the radiotherapy unit of Regional Cancer Institute, Pt. B.D. Sharma University of Health Sciences, Rohtak, Haryana, during the period of January 2007 to October 2009. Total 186 patients with squamous cell carcinoma of oral cavity were analyzed in the study. Based on treatment procedures patients were divided into three groups, group I were under radiotherapy, group II under chemotherapy and group III were of radio chemotherapy together. Clinical isolates from blood and oral cavity were identified by following general microbiological, staining and biochemical methods. The absolute neutrophile counts were done by following the standard methods.

**Results:**

Prevalent bacterial pathogens isolated were *Staphylococcus aureus*, *Escherichia coli*, *Staphylococcus epidermidis*, *Pseudomonas aeruginosa*, *Klebsiella pneumonia*, *Proteus mirabilis*, *Proteus vulgaris* and the fungal pathogens were *Candida albicans*, *Aspergillus fumigatus*. The predominant gram negative bacteria, *Pseudomonas aeruginosa* and *Klebsiella pneumonia* were isolated from blood of radiotherapy and oral cavity of chemotherapy treated cases respectively. The predominance of gram positive bacteria (*Staphylococcus aureus* and *Staphylococcus epidermidis*) were observed in blood of chemotherapy, radio chemotherapy cases and oral cavity of radiotherapy, radio chemotherapy treated cases. Our study also revealed the presence of *C. albicans* fungi as most significant oral cavity pathogens in radiotherapy and radio chemotherapy cases.

**Conclusion:**

Gram positive bacteria and Gram negative were reported from the blood of all the three groups of patients. Oral mucositis played a significant role in oral cavity infection and make patients more prone to *C. albicans* infection.

## Background

Cancer patients remain at substantial risk for developing serious infections despite significant advances in cancer therapy and supportive care. The treatment of malignant conditions with cytotoxic chemotherapy and radiation therapy has become increasingly effective, but it is associated with significant side affects, including toxicities to haemopoietic and non-haematopoietic tissues. Similarly neutropenia is still the most common pre-disposing factor, it is often superimposed on other immunological deficits (e.g. impaired cellular or humoral immunity) each of which is associated with a specific spectrum of infection. Bacterial infections predominate during the early phases of a neutropenic episode, whereas fungal infections occur more often in patients with prolonged neutropenia [1,2]. Beside neutropenia the chemotherapeutic agents and therapeutic radiation also disrupt the mucosal banner of the mouth, leading to severe oral mucositis, gingivitis, oral candidiasis, cellulitis and viral mucosal eruptions [3-10]. The oral mucositis or inflammation of the oral mucosa is painful and is characterized by erythema, edema, and mucosal shedding, which can lead to ulceration and secondary infection [11,12]. Moreover, the oral cavity infections in cancer patients usually result from the combination of neutropenia and mucositis. In oral cavity infection the oral micro flora may be subsequently replaced by potentially pathogenic microorganisms, such as *Candida* sp., (from 72% to 92%), *Candida* carriage was reported common in cancer patients, with *C. albicans* being the predominant species in patients who undergo radiotherapy for Head and neck [13-17]. Oral colonization (up to 93%) and infection (up to 30%) are frequently noted in the patients [18]. The main reason is that the irradiation-induced histological changes leading to oral mucositis, together with salivary quantitative and qualitative changes, have been reported to facilitate yeast growth [13,19]. Beside that a possible explanation for the higher predisposition of irradiated patients to candidosis is due to reduced phagocytic activity of salivary granulocytes against these micro-organisms [20].

Similar to oral cavity infection, bloodstream infections (BSI) also remain serious complications in patients receiving antineoplastic therapy [21]. Bloodstream infections (BSIs) occur due to the failure of the immune system and consequently disseminated the disease. The frequency of BSI infections, their epidemiology, and the invading organisms have changed in parallel with the evolution of medical care, particularly with the emergence of an increasingly ill and immunocompromised population of hospitalized patients who are often heavily dependant on medical support and indwelling devices [22,23]. Currently, slightly more than 50% of BSIs are hospital acquired [24-28].

So, because of a weakened line of defense in oral cancer patients, the present prospective cohort study was carried out, with the aim of isolation, and identification of bacterial, fungal colonization from oral cavity and blood of radiotherapy, chemotherapy and radio chemotherapy treated patients. It is also important to be mentioned that our study was based on evaluation of colonization of fungal species only and did not consider the clinical aspects of the fungal species.

## Methods

### Study design

The present prospective cohort analysis was conducted on the patients undergoing treatment in the radiotherapy unit of Regional Cancer Institute, Pt. B.D. Sharma University of Health Sciences, Rohtak, Haryana, during the period of January 2007 to October 2009. A total of 186 patients with squamous cell carcinoma of oral cavity were analyzed in the study. The present study was approved by Human Ethical Committee of the University (M.D. University, Rohtak) and written consent was also taken from the patients.

### Patient’s population

The patients were divided into three groups depending on their treatment protocol; each category was having 62 cases. First group patients were radiotherapy treated (RT) only (total dose of radiotherapy ranging from 51 to 60 grays in dose of 200cGY/day, 5 days a week), second group patients were given chemotherapy treated (CT) only (3 or 4 Courses of carboplatin, 5-FU, docetaxel/methotrexate/cisplatin given after 21 days gap) and third category patients were given radio chemotherapy simultaneously (RCT) (Between irradiation, chemotherapy courses of paclitaxel, carboplatin and 5-FU given).

### Inclusion criteria

About 10^8^ CFU/ml of bacteria and 10^5^ CFU/ml of fungi cells were considered as pathogenic for the study. The main predisposing factors that can cause oral cavity and blood stream infection in the three studied groups were following:

Bloodstream infection (BSI): BSIs were defined, as isolation of a recognized pathogen (aerobic bacteria and fungi) from one or more blood cultures (BCs) that were unrelated to an infection at another site with, or without fever or hypotension [29,30].

Oral infection: Oral infection was defined as, isolation of recognized pathogens (aerobic bacteria and fungi) from one or more oral swab.

Episodes of BSI: Since any given patients could have a BSI more than once, so we use the term episode of BSI for each separate event [29,30].

Episode of bactermia: The isolation of one bactermia (unimicrobial) or more (polymicrobial) microorganisms in the same blood culture or in a separate blood culture obtained within 24–48 hours [29,30].

Neutropenia: Neutropenia was defined as, an ANC of less than 500 neutrophil/μL that may increase susceptibility to infection [29. 30].

Fever: Oral temperature of ≥ 38.5°C or more within a 24-hours period after initiation of therapy [29,30].

Anemia: A pathologic deficiency for oxygen-carrying hemoglobin in the red blood cells. In case of males Hb. < 14 g/dl and in case of females Hb. < 12 g/dl were considered as anemic cases [31].

Community acquired infection: Any infection acquired before, or within 48 hours of admission to hospital and which was not related to any hospital procedure [29,30].

Nosocomial Infection: Nosocomial infection was defined as, at least one blood culture positive for significant pathogens in patients before, or within 48 hours of admission to hospital [29,30].

Catheter related infection: Infection was considered catheter related when at least one of following conditions were, meet (1) Isolation of same pathogens from catheter tip and blood. (2) Isolation of pathogens from a blood culture obtained from the catheter, but not from another blood obtained from peripheral vein at the same time [29,30].

Oral mucositis: WHO describe oral mucositis into 4 categories, like: grade 0- no change; grade 1 soreness/erythema; grade 2 erythema, ulcers, can eat solids; grade 3 ulcers, requires liquid diet only; grade 4 alimentation not possible [30].

### Exclusion criteria

Patients were excluded from the study if they had clinical or microbiological evidence of bloodstream infection of unknown origin. Patients who developed, fever within 24 hours after administration of chemotherapy and fever subsided within next 24 hours after completion of chemotherapy were also excluded from study. Common skin isolates, including *Coryneforms* and *Bacillus* species excluded from analysis. Coagulase negative *Staphylococci* (CoNS) were only considered as causative pathogens if two or more blood samples drawn on separate occasions showed the growth of the pathogen.

### Clinical and laboratory data

The data on patient’s age, sex, underlying cancer, clinical stage of cancer, medications (antibiotics, cytotoxic drugs), fever, and exposure to radiotherapy or chemotherapy were recorded over the preceding 30 days and an invasive procedure performed over the proceeding 10 days. For every febrile episode of oral infection and blood cavity infection, the data on: date of onset, date of admission, sources of infection, presence of venous catheters and period of their insertion, result of complete blood count, severity and duration of neutropenia were collected.

### Oral cavity specimen handling

Before antibiotics were started, Oral swab were taken by gently rubbing a sterile cotton swab over the labial mucosa, tongue and cancerous lesion [32]. The swabs were incubated in sheep blood agar, saboured dextrose agar, macconkey agar, nutrient agar, and other selective media for primary isolation of the pathogens. These plates were than aerobically incubated for 24–48 hours at 37°C temperature for bacterial pathogens isolation and for 24–72 hours at 30°C in B.O.D. incubator for fungal species isolation.

### Blood Specimen handling

Before antibiotics were started, blood samples (5 ml each) for cultures were obtained from each patient who developed, fever within 21 days following radiotherapy, chemotherapy and radio chemotherapy. One samples isolated from central venous catheter (if present) and other from peripheral vein. Blood cultures were drawn with a sterile system after a sterile pad was placed below the catheter hub and the hub was disinfected with 10% povidone–iodine. Blood samples were than transferred in culture bottles of brain heart infusion broth. Bottles were incubated at 37°C for 7 days. Simultaneously bottles showing positive growth index from blood culture were gram stained and sub cultured on sheep blood agar, saboured dextrose agar, macconkey agar and nutrient agar, simmon citrate agar and cetrimide agar plates. These plates were than aerobically incubated for 24–48 hours at 37°C temperature for bacterial pathogens isolation and for 24–72 hours at 30°C in B.O.D. incubator for fungal species isolation.

### Microbial identifications

The bacterial pathogens were identified after appearance of growth on sub cultured, plates of blood and oral swab by standard microbiological and biochemical procedures. These biochemical tests include: Carbohydrates fermentation tests, urease tests, oxidase test, haemolysis of blood, catalase test, motility tests and growth pathogens on specific media etc. A preliminary examination of fungal colony on SDA was done through gram stained, smear, formation of germ tube, study of micro morphology, morphology on KOH stained smear, assimilation of carbon and nitrogen [33-36].

All isolated pathogens were compared with MTCC standard strains like *S. aureus* with MTCC 96 strain, *S. epidermidis* MTCC 435 strain, *P. vulgaris* MTCC 426 strain, *P. mirabilis* MTCC 425 strain, *E. coli* MTCC 443 strain, *K. pneumonia* MTCC 109 strain, *P. aeruginosa* MTCC 741 strain, *C. albicans* 3017 strain and *A. fumigatus* 2550 strain.

### Absolute neutrophils count

The absolute neutrophils count (ANC) was done by multiplying the total WBC count by percentage of neutrophils (segmented + band) [37].

(1) ANC = WBC × percentage of neutrophils

On the basis of ANC the patients were divided into two categories:

Neutropenia: When ANC was less than 500 (severe risk of infection).

Non neutropenia: When ANC was more than 500 (moderate risk of infection).

### Basic statistical methods

Means values were reported ± standard deviation (SD). Continuous variables mean values were compared by ‘t’ tests. For independent samples difference in proportion of two groups were compared by chi – square test (with Yates correction) or Fisher’s exact test, when appropriate. All test of significance were two tailed. Alpha was set at 0.05. For the logistic regression odd ratio with 95% confidence interval (CI_95_) were calculated. Univariate analysis of dichotomous and ordinal variables was performed by using the procedures for matched data seta in the EpiInfo computer Programme (Epi 6.03: centre for disease control and prevention, USA). Conventional statistical methods were used to calculate means and standard deviation with the help of Microsoft excel 2007.

## Results

### Patients and their characteristics

In this study a total of 186 cases of oral squamous carcinoma divided into three groups have been taken into account. Group I patients had a median age of 60 years (range 25–83 years), with male- female ratio 45:17. Group II patients were having a median age of 45 years (range 30–65 years) with male -female ratio 47:15, and Group III patients were of median age of 50 years (range 48–65 years) with male -female ratio 50:12. Patients in all three groups were mainly of III^rd^ and IV^th^ clinical stage of cancer with predominant underlying disease carcinoma on base of tongue (Table 1).

**Table 1:** Clinical and demographic data of 186 patients (62 cases per group)

**Group I**	
-Age in years, (median range)	60 (25–83)
-Underlying disease	
Carcinoma of oropharynx	12 (19.3%)
Carcinoma of floor of mouth	14 (22.5%)
Carcinoma of base of tongue	17 (27.4%)
Carcinoma of Tonsil Rt. Side	10 (16.1%)
Carcinoma of last molar tooth	9 (14.5%)
-Stage of cancer	
IInd	14 (22.5%)
IIIrd	17 (27.4%)
IVth	31 (50%)
-Male : Female ratio	45:17
Group II	
-Age in years, (median range)	45(30–65)
-Underlying disease	
Carcinoma of oropharynx	0
Carcinoma of floor of mouth	16 (25.8%)
Carcinoma of base of tongue	22 (35.4%)
Carcinoma of Tonsil Lt. Side	7 (11.29%)
Carcinoma of Larynx	17 (27.4%)
-Stage of cancer	
IInd	12 (19.3%)
IIIrd	25 (40.3%)
IVth	25 (40.3%)
-Male : Female ratio	47:15
Group III	
-Age in years, (median range)	50 (48–65)
-Underlying disease	
Carcinoma of oropharynx	13 (20.9%)
Carcinoma of floor of mouth	19 (30.64%)
Carcinoma of base of tongue	30 (48.3%)
Carcinoma of Tonsil Rt. Side	0
Carcinoma of last molar tooth	0
-Stage of cancer	
IInd	10 (16.1%)
IIIrd	22 (35.4%)
IVth	30 (48.3%)
-Male : Female ratio.	50:12

### Oral cavity infection

Predisposing factors of oral cavity infection in three groups have been shown in Table 2. In group 1 out of 62 patients, 35 (43.75%) clinical febrile episodes were found in 22 neutropenic and 45 (52.32%) from 40 non-neutropenic. The most significant risk factors of oral infection were found oral mucositis (grade 4) (P < .05). In case of group II, between 33 neutropenic cases, 46(52.8%) febrile episodes of oral infection were isolated and from 29 non-neutropenic patients, 41(47.12%) episodes were isolated. And the prevalent significant predisposing factor was oral mucositis grade 3 (P < .05). In case of group III, between 44 neutropenic cases, 67 (63.8%) episodes of oral infection were isolated and from 18 non-neutropenic patients, 38 (36.19%) episodes were recovered and the most significant risk factor was mucositis grade 4 (P < .001).

**Table 2:** Predisposing factors for oral cavity infection in febrile episodes of neutropenic and non- neutropenic patients in radiotherapy, chemotherapy and radio chemotherapy treated cases

**Causative factors**	**Group = 1**	**Group = II**	**Group = III**
	**ANC**	**ANC**	**ANC**
	**<500 ≥500**	**<500 ≥500**	**<500 ≥500**
Duration of neutropenia >7	4(6.55) 4(4.87)	4(5.06) 4(7.01)	4(4.34) 6(7.22)
	P = 0.72	P = .71	P = 0.52
Oral mucositis (grade 0)	3(4.91) 3(3.65)	2(2.53) 4(7.01)	5(5.43) 2(2.40)
	P = 0.70	P = .23	P = .44
Oral mucositis (grade 1)	4(6.55) 3(3.65)	3(3.79) 5(8.77)	7(7.60) 6(7.22)
	P = 0.45	P = 0.27	P = .84
Oral mucositis (grade 2)	5(8.19) 5(6.09)	4(5.06) 5(8.77)	9(9.78) 4(4.81)
	P = .74	P = 0.49	P = .33
Oral mucositis (grade 3)	8(13.11) 8(9.75)	19(24.05) 5(8.77)	11(11.95) 7(8.43)
	P = 0.71	P = 0.05*	P = .60
Oral mucositis (grade 4)	9(14.75) 25(30.48)	11(13.92) 13(22.80)	32(34.78) 12(14.45)
	P < .05*	P =0.26	P < .001***
Anemia	4(6.55) 3(3.65)	5(6.32) 3(5.26)	5(5.43) 4(4.81)
	P = .45	P = 1	P = 1
Central venous line present	6(9.83) 7(8.53)	9(11.39) 7(12.28)	7(7.60) 15(18.07)
	P = .97	P = .91	P = .06
Peripheral line present	5(8.19) 3(3.65)	7(8.86) 5(8.77)	4(4.34) 9(10.84)
	P = .28	P = 77	P = .17
Community acquired	6(9.83) 17(20.73)	6(7.59) 3(5.26)	3(3.26) 9(10.84)
	P = 0.12	P = .73	P = .09
Nosocomial acquired	7(11.47) 4(4.87)	9(11.39) 3(5.26)	5(5.43) 9(10.84)
	P = .20	P = .34	P = 0.29

Note: % had been shown in parenthesis, the value in table shows number of patients, ANC = absolute neutrophil count, ANC of < 500 neutrophil/micro L (neutropenic), ANC ≥ 500 neutrophil/micro L (non-neutropenic). P = Probability value with an ANC of <500 neutrophil/micro L (neutropenic) vs. those with ANC > 500 neutrophil/micro L (non-neutropenic). P < .05 * less significant, P < .01 ** significant, P < .001 *** highly Significant.

The pathogens (%) isolated from oral cavity of all three groups have been shown in Tables 3. In group 1 out of 80 episodes, 143 pathogens were recovered, 136 pathogens were from 87 episodes of group II, and 175 pathogens were recovered from 105 episodes of group III.

**Table 3:** Predominant pathogens isolated from oral cavity of neutropenic and non-neutropenic patients treated with radiotherapy, chemotherapy and radiochemotherapy (Group I, II, III)

**Pathogens**	**ANC (<500)ANC(>500)**				**ANC(<500)ANC(>500)**				**ANC(<500)ANC(>500)**			
	**n = 61**	**n = 82**	**OR (95 % C.I)**	**P**	**n = 79**	**n = 57**	**OR (95 % C.I)**	**P**	**n = 92**	**n = 83**	**OR (95 % C.I)**	**P**
(A)Gram + ive	13(21.3)	12(14.6)	1.58(0.61-4.0)	0.41	25(31.6)	18(31.5)	1.00(0.45-2.2)	0.85	39(42.3)	31(37.3)	1.23(0.6-2.3)	0.59
*S. aureus*	8(13.1)	6(7.3)	1.60(0.25-10.7)	0.85	18(22.7)	11(19.2)	1.64(0.38-7.2)	0.67	29(31.5)	15(18)	3.09(1.0-9.6)	<0.05*
*S. epidermidis*	5(8.1)	6(7.3)	0.63(0.09-4.0)	0.85	7(8.8)	7(12.28)	0.61(0.14-2.2)	0.67	10(10.8)	16(19.2)	0.32(0.1-0.9)	<0.05*
(B)Gram -ive	20(32.9)	18(21.9)	1.25(0.57-2.7)	0.68	43(54.4)	33(57.8)	0.87(0.41-1.8)	0.82	22(23.9)	29(34.9)	0.59(0.2-1.1)	0.15
*E. coli*	4(6.5)	6(7.3)	0.50(0.09-2.6)	0.46	7(8.8)	8(14.3)	0.61(0.17-2.1)	0.56	6(6.5)	8(9.6)	0.98(0.2-4.0)	.77
*P. aeruginosa*	3(4.9)	4(4.8)	0.62(0.09-4.1)	0.68	5(6.3)	4(7)	0.95(0.20-4.7)	1.00	5(5.4)	7(8.4)	0.92(0.2-4.0)	0.82
*K. pneumonia*	5(8.1)	5(6.0)	0.87(0.16-4.5)	1	27(34.1)	13(22.8)	2.60(0.93-7.3)	< 0.05*	4(4.3)	5(6)	1.07(0.2-5.5)	1
*P. mirabilis*	5(8.1)	3(3.6)	3(0.41-26.4)	0.40	4(5.0)	6(10.5)	0.46(0.10-2.1)	0.31	4(4.3)	5(6)	1.07(0.2-5.5)	1
*P. vulgaris*	3(3.8)	0	UN	0.23	0	2(3.5)	0.00(0.00-3.14)6	0.18	3(3.2)	4(4.8)	0.99(0.1-6.1)	1
(C) Fungi	28(45.9)	52(63.4)	0.49(0.24-1.0)	< 0.05*	11(13.9)	6(10.5)	1.38(0.43-4.5)	0.74	31(33.6)	23(27.7)	1.33(0.6-2.6)	0.48
*C. albicans*	28(45.9)	40(48.7)	1.30 (1.12-1.5)	< . 001***0	7(8.8)	6(10.5)	0.00(0.00-2.8)	0.23	26(28.2)	23(27.7)	0.00(0.0-1.5)	<.05*
*A. fumigatus*	0	12	UN	.02	4(5.0)	0	UN	1.0	5(5.4)	0	UN	1

Note: n = number of pathogens, UN: Undefined, % had been shown in parenthesis, ANC = absolute neutrophil count, ANC of <500 neutrophil/micro L (neutropenic), ANC >500 neutrophil/micro L (non-neutropenic). P = Probability value with an ANC of <500 neutrophil/micro L (neutropenic) vs. those with ANC >500 neutrophil/micro L (non-neutropenic). P < .05 * less significant, P < .01 ** significant, P < .001 *** highly significant.

The prevalent pathogens isolated from all three different groups have been shown in Table 3. In group 1 *C. albicans* was the most prevalent and significant fungi isolated from oral cavity after radiotherapy (P < .001). Gram positive bacteria were isolated from 6 (27.27%) febrile episodes, in neutropenic patients (13, 21.31%) and in non-neutropenic patients (12, 14.63%). All gram negative bacteria (*E. coil*, *P. aeruginosa*, *K. pneumonia*, *P. mirabilis* and *P. vulgaris*) were isolated in almost in same number in oral infection. The polymicrobial episodes in oral infection were recovered in neutropenic cases (20, 57.14%) and in non neutropenic cases (5, 11.11%). In group II the oral infection causing pathogen was gram-negative bacteria (neutropenic 54.43% and non-neutropenic 57.89%) recovered from 20 neutropenic and 16 non-neutropenic febrile episodes. *K. pneumonia* was most prevalent and significant gram negative bacteria (P < .05). In gram positive bacteria *S. aureus* was the prevalent in neutropenic (18, 22.78%) and nonneutropenic cases (11, 19.29%). The pathogenic fungi were isolated from neutropenic (11, 13.92%) and non-neutropenic (6, 10.52%) patients. The polymicrobial infection was recovered from 14 episodes (30.4%) of 8 (24.24%) neutropenic cases and from 11 (26.8%) episodes of 11 (37.9%) non-neutropenic cases. In group III, 175 pathogens were isolated from 105 febrile episodes. The predominant isolated pathogens were gram positive bacteria recovered, 39 (42.39%) from 28 (53.84%) episodes of neutropenic and 31 (37.34%) pathogens from 15 (51.72%) episodes of non-neutropenic patients. The prevalent gram positive bacteria were *S. aureus* (P < .05) followed by *S. epidermidis* (P < .05). The gram-negative bacteria were isolated in higher amount from non-neutropenic (29, 34.93%) as compared to neutropenic cases (22, 23.91%). All gram negative pathogens were isolated were same in number. *C. albicans* was significant isolated fungi (P < .05) recovered from neutropenic (33.69%), non-neutropenic (27.71%) cases. The Polymicrobial pathogens were isolated from 15 (22.38%) episodes and 9 (23.6%) episodes from non-neutropenic patients.

### Blood streams infection

Predisposing factors for blood infection have been shown in Table 4. In group I out of 62 patients, 35 (43.75%) clinical febrile episodes were found in 22 neutropenic and 45 (52.32%) from 40 non-neutropenic. The most significant risk factors were found oral mucositis (grade 4) (P < .01), followed by community acquired infection (P < .05). In case of group II, between 33 neutropenic cases, 46 (52.8%) febrile episodes of bloodstream infection were isolated and from 29 non-neutropenic patients, 41(47.12%) of febrile episodes were isolated and no significant risk factor was observed. In case of group III, out of 44 neutropenic cases, 67 (63.8%) febrile episodes and out of 18 non-neutropenic patients, 38 (36.19%) of febrile episodes were recovered. The most prevalent significant risk factor was nosocomial acquired infection (P < .001), followed by mucositis grade 4 (P < .001) and central venous line (P < .05).

**Table 4:** Predisposing factors for bloodstream infection in febrile episodes of neutropenic and non- neutropenic patients in radiotherapy, chemotherapy and radio chemotherapy treated cases

**Causative factors**	**Group = 1**	**Group = II**	**Group = III**
	**ANC**	**ANC**	**ANC**
	**<500 ≥500**	**<500 ≥500**	**<500 ≥500**
Duration of neutropenia >7	5(7.81) 4(4.70)	6(6.6) 4(7.27)	10(8.92) 6(6.31)
	P = 0.49	P = 1	P = 0.65
Oral mucositis (grade 0)	5(7.81) 3(3.52)	5(5.55) 4(7.27)	5(4.46) 2(1.78)
	P = 0.28	P = .73	P = .45
Oral mucositis (grade 1)	6(9.37) 3(3.52)	2(2.22) 5(9.09)	7(6.25) 6(6.31)
	P = 0.17	P = 0.10	P = .78
Oral mucositis (grade 2)	4(6.25) 5(5.88)	4(4.44) 5(9.09)	12(10.71) 4 (4.21)
	P = 1	P = 0.30	P = .13
Oral mucositis (grade 3)	5(7.81) 8(9.41)	9(10) 6(10.90)	15(13.39) 7(7.36)
	P = 0.78	P = 0.91	P = .23
Oral mucositis (grade 4)	7(10.93) 25(29.41)	11(12.22) 12(21.81)	32(28.57) 12(12.63)
	P < .01**	P =0.19	P < .001***
Anemia	4(6.25) 3(3.52)	10(11.11) 3(5.45)	9(8.03) 6(6.31)
	P = .46	P = .37	P = .83
Central venous line present	8(12.5) 7(8.23)	15(16.6) 5(9.09)	7(6.25) 15(15.78)
	P = .56	P = .30	P = .05*
Peripheral line present	5(7.81) 3(3.52)	9(10) 5(9.09)	4(3.57) 10(10.52)
	P = .02	P = 91	P = .08
Community acquired	6(9.37) 20(23.52)	6(6.06) 2(3.63)	6(5.35) 11(11.57)
	P < .05*	P = .71	P = .17
Nosocomial acquired	9(14.06) 4(4.70)	13(14.44) 4(7.27)	5(4.46) 16(16.84)
	P = .08	P = .29	P < .001***

Note: % had been shown in parenthesis, the value in table shows number of patients, ANC = absolute neutrophil count, ANC of <500 neutrophil/micro L (neutropenic), ANC ≥500 neutrophil/micro L (non-neutropenic). P = Probability value with an ANC of <500 neutrophil/micro L (neutropenic) vs. those with ANC >500 neutrophil/micro L (non-neutropenic). P < .05 * less significant, P < .01 ** significant, P < .001 *** highly Significant.

**Table 5:** Predominant pathogens isolated from blood of neutropenic and non-neutropenic patients treated with radiotherapy, chemotherapy and radio-chemotherapy (Group I, II. III)

**Pathogens**	**ANC (<500)ANC(>500)**				**ANC(<500)ANC(>500)**				**ANC(<500)ANC(>500)**			
	**n = 64**	**n = 85**	**OR (95 % C.I)**	**P**	**n = 90**	**n = 55**	**OR (95 % C.I)**	**P**	**n = 112**	**n = 95**	**OR (95 % C.I)**	**P**
(A)Gram + ive	23(35.9)	18(21.1)	2.09(0.95-4.6)	0.07	52(5.7)	25(45.4	1.64(0.7-3.4)	0.20	61( 54.4)	35(36.8)	2.0(1.1-3.7)	< 0.01**
*S. aureus*	15(23.4)	12(14.1)	0.94(0.21-4.1)	0.81	42(4.6)	11(2)	5.35 (1.6-17.6)	< .001**	21( 18.7)	28(29.4)	0.1(0.0-0.3)	< .001***
*S. epidermidis*	8(12.5)	6(7.0)	1.07(0.24-4.7)	0.81	10(1.1)	14(25.4)	0.19(0.0-0.6)	< .001**	40(35.7)	7(7.3)	7.6(2.6-23.1)	< .001***
(B)Gram -ive	26(40.6)	56(65.8)	0.35(0.1-0.7)	< 0.001***	24(2.6)	22(4)	0.55(0.2-1.1)	0.13	39(34.8)	42(44.2)	0.67(0.3-1.2)	0.21
*E. coli*	10(15.6)	9(10.5)	0.34(0.1-0.9)	0.04	7(7.7)	6(10.9)	1.10(0.2-4.7)	0.85	11(9.8)	17(17.8)	0.58(0.2-1.6)	0.35
*P. aeruginosa*	6(9.3)	28(32.9)	0.30(0.0-0.9)	< 0.05*	7(7.7)	7(12.7)	0.88(0.2-3.7)	0.90	12(10.7)	12(12.6)	1.11(0.3-3.2)	0.97
*K. pneumonia*	5(7.8)	14(16.4)	0.71(0.1-2.5)	0.76	6(6.6)	5(9.0)	1.13(0.2-5.4)	0.86	10(8.9)	7(7.3)	1.72(0.5-5.8)	0.47
*P. mirabilis*	5(7.8)	5(5.8)	2.43(0.5-11.1)	0.27	4(4.4)	3(5.4)	1.27(0.2-8.4)	1.00	4(3.5)	5(5.2)	0.85(0.1-4.0)	1
*P. vulgaris*	0	0	0	0	0	1(1.8)	0.00(0.0-16.4)	0.47	2(1.7)	1(1.0)	1.84(0.1-53.7)	1
(C) Fungi	15(23.4)	11(12.9)	2.06(0.8-5.2)	0.14	14(15. 5)	8(14.5)	1.20(0.4-3.3)	0.88	12(10.7)	18(18.9)	0.51(0.2-1.2)	0.13
*C. albicans*	9(14.0)	6(7.0)	1.25(0.2-8.0)	1.00	8(8.8)	5(9.0)	0.80(0.0-6.5)	1.00	7(6.2)	10(10.5)	1.12(0.2-6.3)	0.82
*A. fumigatus*	6(9.3)	5(5.8)	0.80(0.1-5.0)	1.00	6(6.6)	3(5.4)	1.25(0.1-10.5)	1.00	5(4.4)	8(8.4)	0. 89(0.1-4.9)	0.82

Note: n = number of pathogens, % had been shown in parenthesis, ANC = absolute neutrophil count, ANC of <500 neutrophil/micro L (neutropenic), ANC > 500 neutrophil/micro L (non-neutropenic). P = Probability value of patients with an ANC of <500 neutrophil/micro L (neutropenic) vs. those with ANC >500 neutrophil/micro L (non-neutropenic). P < .05 * less significant, P < .01 ** significant, P < .001 *** highly significant.

The pathogens (%) isolated from blood of all three groups have been shown in Table 4. During the study a total of 149 pathogens were recovered from 80 febrile episodes of BSI from group I, 145 pathogens from 87 febrile episodes from group II and 207 pathogens were recovered from 105 febrile episodes in group III.

In group I gram negative bacteria were recovered from 16 (57.14%) febrile episodes of patients, in neutropenic 26 (40.62%) and 56 (65.80%) in non-neutropenic patients (P < .001). *P. aeruginosa* was the most significant (P < .05) gram negative bacterium. Gram positive bacteria were isolated from, 8 (28.5%) febrile episodes, 23 (35.93%) in neutropenic patients and 18 (21.17%) in non-neutropenic patients. *C. albicans* was predominant in neutropenic 9 (14.06%) and nonneutropenic 6 (7.05%) patients. Out of all febrile episodes of neutropenic and non- neutropenic, 7 episodes (20%, 15.5%) were polymicrobial. In case of group II the main pathogens were gram-positive bacteria isolated in 25 (45.45%) febrile episode of neutropenic (57.77%), and 26 (40%) febrile episode of non-neutropenic 35 (62.85%) patients. Gram-negative bacteria were isolated, 24 (26.26%) from 10 (25%) febrile episodes in neutropenic and 22 (40%) from 9 (25.71%) febrile episode in non-neutropenic patients. Pathogenic fungi isolated from neutropenic and non-neutropenic patients were 14 (15.5) and 8 (14.54%). A total of 5 cases (15.15%) in 6 episodes (13.04) were polymicrobial in neutropenic patients and 4 cases (13.7%) in 6 episodes (14.6%) were in non-neutropenic cases. In case of group III 207 pathogens were isolate from 105 febrile episodes. Out of 37 febrile episodes of neutropenic and 17 febrile episodes of non neutropenic cases the most significant isolated pathogen (P < .01) were gram-positive bacteria. The gram-negative bacteria were found higher in nonneutropenic cases (42, 44.21%) as compared to neutropenic cases 39 (34.82%). The fungi isolated from neutropenic patients, 10.71% and 18.94% from non-neutropenic patients. The polymicrobial BSI infection was reported from 16 episodes (23.8%) of neutropenic and 12 episodes, (31.5%) of non neutropenic patients.

## Discussion

This prospective cohort study is first from Haryana, India and is based on evaluation of the rate, risk factors and outcomes of treatment procedures between oral cancer patients. The present report describes colonization of bacterial and fungal infectious pathogens in oral cancer patients**.**

The colonization of microorganisms in cancer patients was found to occur in oropharynx as well as gastrointestinal system, urinary system and airways. Colonization starts within 48 hours of hospitalization [38]. Number of studies proved that Neutropenia increases the microbial colonization [39-41]. Our observation also illustrates that the colonization of microorganisms was higher in blood and oral cavity of neutropenic cases after chemotherapy, radio chemotherapy.

Nowadays, in most of hospitals, there is a shift of the microbial spectrum of cancer patients from gram-negative to gram positive, compared with the predominance of gram-negative species in the 1960s and 1970s [42-46]. Nevertheless in developing countries there is a different situation where the predominant pathogens are gram-negative, the reason maybe the people cannot afford to give routinely prophylactic oral antibiotics, such as quinolones, and use less central lines. The predominance of gram negative bacteria in developing countries can also be explained with the help of various studies. A study was conducted on febrile neutropenic patients in a hospital from Lebanon and observed that the gram-negative bacteria were responsible for 78.8% (26/33) of bloodstream infections compared to 33.3% (11/33) gram-positive organisms. In the present study dominant gram negative bacteria were of *E. coli* and *P. aeruginosa*. A possible explanation of the observed high incidence of gram-negative infections in Lebanon was the relatively low proportion of indwelling catheters [47]. Another study was carried in Malaysia observed that out of 120 episodes, 60.02% were gram-negative organisms of Enterobacteriaceae [48]. A similar pattern of predominant gram-negative bacteria (61%) was seen in a study of hematologic malignancy patients from Brazil [49]. Similar to above study we have also observed that main pathogen isolated from blood of group I and oral cavity of group II were of gram negative bacteria. This may be due to absence of use of catheter as a routine practice during the period of our analysis.

In our study *P. aeruginosa* was the main gram negative bacteria in blood stream and *K. pneumonia* was in oral cavity. Similar to our study the presence of *P. aeruginosa* as an infectious pathogen was also observed by Raje et al., [50]. They reported *P. aeruginosa* (28%) as a major pathogen in febrile neutropenic patients of acute lymphobalastic leukemia cases. Karim et al., [51] and Saghie et al., [52] were also observed presence of *P. aeruginosa* in 31% and 38% respectively from febrile neutropenic cases. However our study showed the proportion of *P. aeruginosa* was higher in non- neutropenic cases.

The present report also revealed the predominance of gram positive bacteria (*S. aureus* and *S. epidermidis*) in blood of group II, III and oral cavity of I, III group. The predominance of gram positive bacteria as infectious pathogen was also proved by other studies did in India. Jagarmuldi et al., [43] conducted a study on acute leukemia cases and observed 38.5% of *S. aureus* infection in 240 febrile episodes and in other study *S. aureus* (39%) infection was observed in blood after chemotherapy [53]. The prevalence of gram positive bacteria may be due to that oral cancer patient were undergone treatment of high intensive chemotherapy, radio chemotherapy which may be led to damage of the mucosal barriers and increases the risk of infection with gram-positive oral (and GI) flora [54]. In favor of that reason, we observed the significant predisposing factor for blood stream infection was the use of central venous line (P < .05) in group III, which may be facilitated the entry of organisms colonizing the skin into the bloodstream, and thus increase the rate of Staphylococcal infections in blood and oral cavity [2,43]. Our study also observed another significant predisposing factor of bloodstream infection in group III was nosocomial acquired (P < .01). The role of nosocomial acquisition of *S. aureus* infection was also demonstrated in various studies did at five centers in Egypt and a provincial hospital in north east Thailand [55,56]. Nosocomial infection of *S. aureus* in developing countries is probably common, the reasons for which may include lack of hand washbasins or hand washing, overcrowding in hospital wards and clinics, lack of infection control training or policies, the inability to isolate specific patients, and lack of diagnostic microbiology facilities [56].

Another salient featured of our study was the colonization of *C. albicans* as most significant oral cavity pathogens in group I and III patients. *C. albicans* was isolated in group I neutropenic, non neutropenic cases in proportion of 45.90%, 48.78% respectively and in group III its proportion in neutropenic, non neutropenic cases was 28.2%, 27.72% respectively. The proportion of C*. albicans* in group I i.e. radiotherapy treated, cases was similar to various studies conducted on oral candidiasis after radiotherapy and showed a wide variation ranging from 17 to 52.5% [4,17,19,57-60]. The colonization of C. albicans in oral cavity of radiotherapy treated, cases may be due to the reason that our patients were often unable to maintain satisfactory oral health and nutritional status during RT, mainly because of low income and educational level. This reasons of colonization of *C. albicans* in oral cavity was also observed in study did in Brazilian patients undergoing head and neck radiotherapy [17]. The pathogenesis of candidal infections is complex encompassing both fungi and host factors. Candidal colonisaion appears to be influenced by adherence mechanisms among fungi and oral epithelial cells. Radiotherapy-induced hyposalvation also encourages oral candidal colonization that often leads to oral candidiasis [19].

We have also observed other reason of colonization of *C. albicans* may be oral mucositis, which played a significant role in oral cavity infection of all three groups. Generally it is also accepted that oral mucositis, is of multifactorial origin, and it is ranged among 20% and 100% in patients receiving different types of cancer treatments [61-65]. Other reason for the prevalence of mucositis in our study may be due to fluorinated 5-fluorouracil (5-FU) which was the most effective and frequently used antineoplastic agent for the treatment of oral cancer. There are others reports which also showed mucositis (4 to 74% for head and neck cancers) induced by 5-FU [66,67].

Some limitations were present in our study, first was that we did not take the clinical features of Oral candidosis in our patients i.e. we were not able to evaluate that whether the Oral candiasis remain confined to the oral cavity, or spread to oesophageal or more widely to cause systemic candidosis. This study also is limited to infections caused by aspergillosis and candidiasis. Although these two pathogens represent most of fungal infections, infections due to other fungal species may result an additional burden to the oral cancer cases.

## Conclusion

Radiotherapy and chemotherapy have been widely used for the treatment of malignant lesions in oral cancer patients to increase the survival rates. However, these therapies are still associated with several adverse reactions that affect patient quality of life significantly, and may even affect the progress of the treatment. Taking into account that the occurrences of oral cancer rates are probably going to be the increase in near future, it is extremely important that health professionals are familiarized with the complications from anti-neoplastic treatments. The present study revealed that radio and chemotherapy treated immunocompromised patients are prone to bacterial and fungal infection with predominance of gram positive bacteria. This study also revealed the presence of *C. albicans* fungi as most significant oral cavity pathogens in radiotherapy and radio chemotherapy treated patients. So the multidisciplinary treatment, including medical team, dental surgeons, speech therapists, nutritionists and psychologists, is the best option to minimize or even prevent such complications in oral cancer cases.

## Competing interests

The authors declare that they have no competing interests.

## Authors’ contributions

All authors have equal contribution in study designs, experiment, data analysis and interpretation of data. Similarly all authors have critically reviewed the manuscript, approved of its contents and consented to its communication for publication.

## Pre-publication history

The pre-publication history for this paper can be accessed here:

http://www.biomedcentral.com/1472-6831/12/22/prepub
